# MicroRNA Regulation of Cholesterol Metabolism

**DOI:** 10.1155/2012/847849

**Published:** 2012-08-05

**Authors:** Noemi Rotllan, Carlos Fernández-Hernando

**Affiliations:** Marc and Ruti Bell Vascular Biology and Disease Program, Leon H. Charney Division of Cardiology, Departments of Medicine and Cell Biology, New York University School of Medicine, New York, NY 10016, USA

## Abstract

Disruption of cellular cholesterol balance results in pathologic processes including atherosclerosis, metabolic syndrome, type II diabetes and Alzheimer's disease. Maintenance of cholesterol homeostasis requires constant metabolic adjustment, achieved partly through the fine regulation of the classical transcription factors (e.g., by SREBP and LXR), but also through members of a class of noncoding RNAs termed miRNAs. Some miRNAs have now been identified to be potent post-transcriptional regulators of lipid metabolism genes, including miR-122, miR-33, miR-758, and miR-106b. Different strategies have been developed to modulate miRNA effects for therapeutic purposes. The promise demonstrated by the use of anti-miRs in human preclinical studies, in the case of miR-122, raises the possibility that miR-33, miR-758, and miR-106b may become viable therapeutic targets in future. This review summarizes the evidence for a critical role of some miRNAs in regulating cholesterol metabolism and suggests novel ways to manage dyslipidemias and cardiovascular diseases.

## 1. Introduction

Cholesterol is the major component of mammalian cells and is essential for diverse cellular functions. Cholesterol levels are maintained through tightly regulated and complex mechanisms. It is well known that insufficient or excessive cellular cholesterol results in a wide range of pathologies, including atherosclerosis, metabolic syndrome, type II diabetes, and Alzheimer's disease (AD) [1–3]. Cholesterol homeostasis has been extensively studied, from the de novo biosynthesis to internalization of exogenous cholesterol, through the efflux of excess cholesterol and finally its elimination through bile. The classical transcription factors that regulate its homeostasis are the sterol response element binding proteins (SREBPs) [4, 5] and the liver X receptors (LXRs) [6, 7].

In addition to the classical transcriptional regulators, a class of noncoding RNAs, termed microRNAs (miRNAs) has emerged as critical regulators of gene expression acting predominantly at the posttranscriptional level. This large family of short (22-nucleotide) noncoding RNA binds to the 3′ untranslated (3′UTR) region of mRNA, thereby repressing gene expression. Thus, they are implicated in the control of many physiological and pathological processes [8–10]. The role of miRNAs in the regulation of lipid metabolism is just beginning to be explored. Several miRNAs have been described to regulate lipid metabolism, including miR-122, miR-33, miR-758, and miR-106b [11–14] (Table 1). Other microRNAs such as miR-370, miR-378/378*, miR-143, miR-27, miR-29a, miR-302a, and miR-335 have also been shown to regulate lipid homeostasis [15–21]. It is also important to highlight that the finding of an exogenous plant microRNA, miR168a, that could bind to the human/mouse low-density lipoprotein receptor adapter protein 1 (LDLRAP1) mRNA, inhibits LDLRAP1 expression in liver and consequently decrease LDL clearance.

This paper addresses recent research and links between miRNAs and their role in regulating cholesterol metabolism and suggests that manipulating their expression *in vivo* may open new avenues for treating dyslipidemias and cardiovascular diseases.

## 2. Cholesterol Metabolism and Its Regulation

Cholesterol is the precursor of steroid hormones, bile acid, and vitamin D and is required for the maintenance of cell membrane fluidity, membrane formation, cell proliferation and embryonic development [26, 27]. An excess of plasma cholesterol leads to its accumulation in the artery wall causing atherosclerosis, the main cause of death in Western societies [28]. Levels of cholesterol are maintained through a tightly regulated and complex mechanism that includes the de novo biosynthesis, internalization of exogenous cholesterol, and efflux of cholesterol excess.

These mechanisms are regulated by transcription factors such as SREBPs and LXRs. SREBPs activate the expression of a variety of genes required for cholesterol, triglycerides, fatty acids, and phospholipid uptake and synthesis. In mammals there are three SREBP isoforms: SREBP1a and SREBP1c encoded by the *Srebp1* gene and SREBP2, encoded by *Srebp2 *gene. SREBP1c regulates the transcription of genes involved in fatty acid metabolism, such as fatty acid synthase FASN [5–7]. On the other hand, SREBP2 and SREBP1a activate the transcription of cholesterol-related genes, including 3-hydroxy-3 methylglutaryl coenzyme A reductase (HMGCR), the rate-limiting enzyme that regulates cholesterol biosynthesis, and low-density lipoprotein receptor (LDLr), that scavenges circulating LDL from the bloodstream [5–7].

In addition to SREBPs, the LXRs also contribute to cholesterol and fatty acid homeostasis. LXRs are activated in response to elevated cholesterol levels (oxysterols) and induce the expression of proteins involved in cholesterol absorption, transport, excretion, and efflux, including the ATP binding cassette transporters A1 (ABCA1), G1 (ABCG1) or G5/G8 (ABCG5/G8), and apolipoprotein E (apoE) [29–31].

## 3. MicroRNAS

miRNAs are small endogenous RNAs approximately 22 nucleotides in length that have emerged as important posttranscriptional regulators of different protein-coding genes. It was first discovered in the nematode *Caenoshabditis elegans*, and since then has been identified in the genomes of most plants, animal, and viruses [8–10, 32].

The miRNAs identified to date are currently curated and annotated at miRBase, hosted by the Sanger Institute as a publicly available repository (http://microrna.sanger.ac.uk/). Experimental approaches using bioinformatic predictions indicates that a single miRNA may simultaneously target more than 100 mRNAs [33]. Similarly, a single mRNA could be regulated by many miRNA. Human miRNAs are predicted to control the activity of 30–60% of all protein-coding genes [32–34]. Thus, their deregulation is closely linked to human diseases, including heart disease and vascular disorders [35–38]. Very interestingly, microvesicles, exosomes, apoptotic bodies, lipoproteins, and large microparticles contain miRNAs. miRNAs are stable in plasma and differential plasma miRNA profiles have been described for many diseases, including fatty liver [39], atherosclerosis [40], and cancer [41–45]. Circulating extracellular miRNAs have enormous potential as novel disease biomarkers. miRNAs could, therefore, be considered like hormones as a possible form of intercellular communication; however, their physiological function and *in vivo* role remains to be definitively established.

## 4. MicroRNA Biogenesis and Function

**Figure 1:**
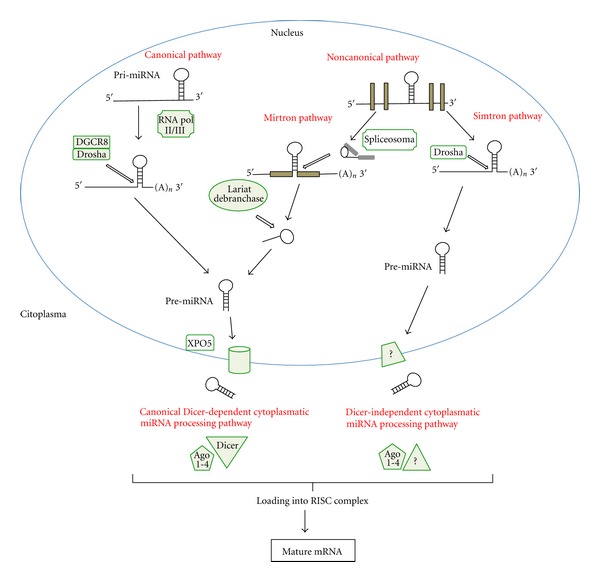
The miRNA biogenesis pathway. MicroRNAs generated by the canonical pathway are transcribed as precursor RNAs from intergenic, intronic, or polycistronic genomic loci by RNA polymerase II (RNA Pol II/III). The primary miRNA (pri-miRNA) transcript forms a stem-loop structure that is processed by the DGCR8/Drosha complex. The pre-miRNA is exported to the cytoplasm by XPO5, where it is processed into a mature miRNA duplex by Dicer. Finally, the mature miRNA enters into the RISC complex in association with an AGO family member. In the noncanonical pathway, mirtrons, a subset of miRNAs derived from introns, are processed by the spliceosome and the debranching enzyme. In addition to mirtrons, another non-canonical pathway called the simtron pathway involves Drosha but not its partner DGCR8 or Dicer. The mature miRNA produced by these different pathways leads to translational repression or degradation of the target mRNA.

The canonical pathway is the classical pathway for the biogenesis of miRNAs. Canonical miRNAs in animals are transcribed in the nucleus by RNA polymerase II, generating a primary long miRNA (pri-miRNA) (Figure 1). These pri-miRNAs are usually hundreds of nucleotides long and contain local hairpin structures. Then, these hairpins are processed sequentially in the nucleus and cytoplasma into a 70-nucleotide hairpin-structure precursor (pre-miRNA) by a multiprotein complex containing a variety of cofactors and two core components, a ribonuclease III (Drosha) and a double-stranded RNA-binding domain protein (DGCR8/Pasha). After that, the pre-miRNA is exported to the cytoplasm by Exportin-5 (XPO5). In the cytoplasm, the pre-miRNA is processed into a ~21–23 nt mature miRNA duplex by the endonuclease Dicer. One of the duplex strands is preferentially loaded into the RNA-induced silencing complex (RISC) in association with an Ago family member producing a functional complex that binds to its RNA target. Thus, miRNAs control gene expression by binding to the 3′UTRs of their targets through Watson-Crick base pairing between the target and the 5′-end of the miRNAs, known as the seed sequence (2–8 nt). This interaction typically leads to the translational repression of target mRNAs by either transcript destabilization, translational inhibition, or both. Recent studies have shown that miRNAs can also repress mRNA targets through binding to other regions, including 5′UTRs or protein-coding exons [46–49] and in some cases may even activate translation [48, 50–52]. However, the mechanistic details of protein synthesis inhibition by miRNAs are not well understood. Potential pathways include sequestration from ribosomes (by relocation into P bodies), blockage of translational initiation, translational repression after initiation, and target deadenylation coupled to transcript degradation [53, 54].

In addition to the classical pathway, an alternative, non-canonical pathway for the biogenesis of some intronic miRNAs (mirtrons) has also been reported. Mirtrons are processed from the host gene by the spliceosome, exported from the nucleus by XPO5, cleaved by Dicer and loaded into the RISC complex. Moreover, another alternative miRNA processing pathway has been recently described (the simtron pathway). The simtron (splicing-independent mirtron-like miRNAs) pathway involves Drosha but does not required Drosha's binding partner DGCR8 or the endonuclease, Dicer [55]. Thus, these miRNA are processed in a manner that does not required splicing, the multiprotein complex, Dicer or Ago2 (Figure 1).

To study the microRNAs functions, antisense reagents against miRNAs have been developed as a reverse genetic tool. Synthetic oligonucleotide analogues, including 2′-O-methyl oligonucleotides [56], locked nucleic acids [57], 2′-O-methoxyethyl oligoribonucleotides [20], and morpholinos [58] have been tested. These antisense nucleotide analogues have been used to knock down miRNAs in cultured cells [20, 56, 57], and in live animals including zebrafish [58], *D. melanogaster* [59], and mice [22].

## 5. Control of Cholesterol Metabolism**** by MicroRNAs

### 5.1. Role of MiR-122 in Liver Metabolism

The liver is the major regulator of cholesterol and lipoprotein metabolism. miR-122 is highly expressed in the liver, and it is estimated to account for approximately 70% of all liver miRNA [22, 60]. miR-122 is highly conserved from human to frogs, suggesting an important role for this miRNA that has been under selective pressure throughout evolution [61]. A role for miR-122 in lipid metabolism was revealed in knockdown studies [11, 23]. miR-122 inhibition by antisense oligonucleotides (ASO) in mice resulted in increased hepatic fatty-acid oxidation and a reduced cholesterol synthesis [23]. In addition, miR-122 inhibition reduced total plasma cholesterol by 25–35%, and this was reflected by changes in both the LDL and HDL fractions [23]. Similar effects were observed in African green monkeys treated with miR-122 antagomirs wherein inhibition caused a dose-dependent decrease in plasma cholesterol without any signs of toxicity [11]. Due to the lack of toxicity, miR-122 has become a strong candidate as a therapeutic target in the treatment of hypercholesterolemia in humans. miR-122 inhibition caused a significant decrease of genes involved in cholesterol synthesis including 3-hydroxy-3-methylglutaryl-CoA synthase 1 (HMGCS1), 3-hydroxy-3-methylglutaryl-CoA reductase (HMGCR), 7-dehydrocholesterol reductase (DHCR7), and squalene epoxidase (SQLE) [22]. Conversely, miR-122 overexpression increases the expression of HMGCS1, DHCR7 and SQLE [22]. Importantly, all these genes are not direct targets of miR-122. Therefore, the mechanism by which miR-122 regulates lipid metabolism remains undetermined. This lack of a mechanistic understanding of the effects of miR-122 on cholesterol homeostasis, and the possibilities of other adverse consequences as the decline levels of HDL, both in mice and in nonhuman primates [11, 23, 62], and hepatocellular carcinoma, has also dampened the enthusiasm for the development of miR-122 antisense technologies as a therapeutic approach for long-term management of cholesterol disorders. It is important to take into account that many miR-122 validated targets are involved in glucose homeostasis and the Krebs cycle, including aldolase A (ALDOA) and citrate synthase (CS) [22, 23]. In addition to regulate lipid and glucose metabolism, miR-122 also plays an important role in regulating iron homeostasis [63]. miR-122 inhibition increased the expression of several genes that control systemic iron levels, such as hemochromatosis (Hfe), hemojuvelin (Hjv), bone morphogenetic protein receptor type 1A (Bmpr1a) and Hamp. Interestingly, mice treated with miR-122 antisense oligonucleotides develop systemic iron deficiency, characterized by reduced plasma and liver iron levels, mildly impaired hematopoiesis, and increased extramedullary erythropoiesis in the spleen.

Recently, miR-122 was found to be required for the propagation of hepatitis C virus (HCV). miR-122 binds two positions in the 5′UTR of the HCV genome, and this binding is essential to viral accumulation and propagation infected hepatocytes [64–66]. In a recent study in nonhuman primates, silencing of miR-122 resulted in a sustained reductions in HVC viremia and improvement in liver pathology, with no evidence of viral resistance [66]. Very interestingly, miR-122 inhibition also decreased Bach1 and increased heme oxygenase-1 (HO-1), which is an antioxidant defense and key cytoprotective enzyme repressed by Bach1 [67]. These data suggest that the therapeutic targeting of miR-122 and upregulation of HO-1 may represent a new strategy for anti-HCV intervention and cytoprotection.

### 5.2. MiR-33: A Key Regulator of Lipid Metabolism

We and others have recently identified *miR-33a *and *miR-33b*, intronic miRNAs located within the *Serbp2* and *Srebp1* genes, respectively [12, 24, 25]. *mir-33a *and *mir-33b* are co-transcribed with their host genes and regulate cholesterol and fatty acid metabolism. miR-33 overexpression strongly represses ABCA1 expression at the RNA and protein level and decreases cellular cholesterol efflux to apolipor protein A-I (ApoA-I), a key step in regulating reverse cholesterol transport (RCT). Conversely, antagonism miR-33 upregulates ABCA1 expression *in vitro* and *in vivo* and promotes cholesterol efflux to ApoA-I. Importantly, *in vivo *inhibition of miR-33 expression leads to a significant increase in plasma HDL levels and the regression of atherosclerosis, thus confirming the physiological effects of miR-33 in regulating lipid metabolism [12, 25, 68, 69].

In addition to ABCA1, two important genes involved in cholesterol metabolism were described as targets of miR-33: ABCG1 which mobilizes cellular free cholesterol to more lipidated HDL particles, and Niemann Pick C1 (NPC1), which transports cholesterol from lysosomes to other cellular compartments. Interestingly, *Abcg1* has two miR-33 binding sites in its 3′UTR that are only present in rodents, suggesting that cellular efflux to mature HDL is differently regulated between species [12, 24]. Another interesting difference between humans and rodents is that the 3′UTR of *Npc1* in humans contains two miR-33 binding sites resulting in a significant repression of NPC1 protein expression, whereas mice only contain one site, which is modestly suppressed by miR-33 [12]. ABCA1 also plays a key role in the biogenesis of HDL in the liver and intestine [31, 70]. To assess whether anti-miRNA-33 therapy increases liver ABCA1 expression and plasma HDL levels, several groups silenced miR-33 expression using a variety of strategies including modified oligonucleotides and antisense oligonucleotides expressed in lentiviral or adenoviral constructs. As expected, mice treated with anti-miR-33 oligonucleotides have a significant increase in liver ABCA1 expression and plasma HDL levels. These results were later confirmed genetically in the miR-33 knockout mice. Although the preclinical studies of miR-33 inhibition in mice are encouraging, extrapolation of these findings to human is complicated by the fact that mice lack miR-33b. Nevertheless, anti-miR-33 therapy in nonhuman primates has demonstrated to be very effective in increasing the levels of HDL and reducing VLDL [71].

miR-33a and miR-33b also target genes involved in the *β*-oxidation of fatty acids, including carnitine palmitoyltransferase 1A (CPT1A), carnitine O-octanoyltransferase (CROT), hydroxyacyl-CoA dehydrogenase/3-ketoacyl-CoA thiolase/enoyl-CoA hydratase (HADHB), 5′ adenosine monophosphate-activated protein kinase (AMPK), and sirtuin 6 (SIRT6). Moreover, miR-33a and miR-33b also target the insulin receptor substrate 2 (IRS2), an essential component of the insulin-signaling pathway in the liver [72]. In addition to the role of miR-33 in regulating cholesterol and fatty acid metabolism, we have also recently shown that miR-33 regulates cell cycle progression and cellular proliferation [73]. miR-33 negatively regulates cyclin-dependent kinase 6 (CDK6) and cyclin D1 (CCND1), which results in cell cycle arrest in G1 phase. Furthermore, this study also shows that *in vivo* inhibition of miR-33 using antisense oligonucleotides improves liver regeneration after partial hepatectomy [73].

Altogether, these data suggest that the inhibition of miR-33 expression may be a promising strategy to treat atherosclerotic vascular disease, metabolic syndrome, and liver regeneration in chronic liver disease.

### 5.3. Role of miR-758 and miR 106b in Regulating Cholesterol Homeostasis

miR-758 and miR-106b also regulate post-transcriptional ABCA1 expression [13, 14]. miR-758 was identified performing an unbiased genomewide screen together with a bioinformatic analysis. miR-758 was downregulated by cellular cholesterol content in macrophages and in the liver from mice fed a high-fat diet [13]. Even though the mechanism by which miR-758 expression is regulated remains unclear, these data strongly suggest that the downregulation of miR-758 in cholesterol-loaded cells may cooperate to up-regulate ABCA1 expression to avoid further cholesterol accumulation [10]. Interestingly, miR-758 is highly expressed in brain tissue and human neuronal cell lines. Overexpression of miR-758 in H4 human neuroglioma cells significantly decreases ABCA1 expression. Moreover, several genes involved in amino acid synthesis, including sodium-coupled neutral amino acid transporter 1 (SLC38a1), neurite outgrowth, such as neurotrimin (NTM), and the development of the nervous system, like ephrin type-A receptor 7 (EPHA7) were also downregulated in H4 cells transfected with miR-758 mimics [13].

Genetic studies with Alzheimer disease (AD) mouse models have demonstrated that the deletion of ABCA1 increases A*β* deposition, while overexpression of ABCA1 dramatically reduces A*β* deposition, suggesting a role for ABCA1 in A*β* metabolism [22, 74–76]. Interestingly, miR106b has been recently reported to decrease ABCA1 expression and impair cellular cholesterol efflux in neuronal cells. Moreover, Neuro2a cells transfected with miR-106b dramatically increase levels of secreted A*β* by increasing A*β* production and preventing A*β* clearance [14]. Overall, these findings suggest an important role for miR-758 and miR106b in regulating neurological functions and might have important implications in the pathogenesis of AD through posttranscriptional repression of ABCA1. Interestingly, miR-33 is highly expressed in the brain, and many predicted targets for miR-33 are involved in neurogenesis, such as Sema-3a and netrin-1, and synaptic regulation, including glutamate receptor ionotropic Kainate 2 (GRIK2) and glutamate receptor ionotropic AMPA 3 (AMPA 3).

## 6. Conclusion

miRNAs represent an elegant layer above transcriptional control for both fine-tuning and dramatically altering activity and output of cell signaling. In addition, miRNAs may serve as points of crosstalk between signaling pathways, by integrating transcriptional inputs or by their functional regulatory output on different pathways. Recently, short interfering RNAs (siRNAs) and miRNAs have gained considerable attention as therapeutic targets. Different strategies have been developed to modulate miRNA effects for therapeutic purposes. Inhibition of miR expression can be achieved using antisense oligonucleotides “antagomirs,” or their chemically modified versions, 2′-O-methyl-group(OMe)-modified oligonucleotides and locked nucleic acids (LNAs) “antimiRs”, as well as by inhibiting the production of the mature forms via affecting their processing. There is tremendous therapeutic potential for the treatment of cardiovascular diseases, by either overexpression or inhibition of miRNAs. The data summarized in this paper pointed out that anti-miR-33, miR-758 therapy, and miR-106 may be useful for treating dyslipidemia and cardiovascular disorders.

## Figures and Tables

**Table 1:** MicroRNAs involved in cholesterol metabolism.

miRNA	Target tissue/cell type	Target genes	Biological function	References
miR-122	Primary mouse hepatocytes	*ALDOA* *CS*	Glucose homeostasis Krebs cycle	[22, 23]

miR-33a and miR-33b	Liver and macrophage	*ABCA1; * *ABCG1*; *NPC1 * *CROT; * *HADHB; CPT1; SIRT6; PRKAA1 * *IRS2 *	Cellular cholesterol efflux Fatty acid oxidation Insulin signaling	[12, 24, 25]

miR-758	Human and mouse macrophages and hepatic cell line	*ABCA1*	Cellular cholesterol efflux	[13]
	Human neuroglioma cell line	*SLC38a1; NTM; EPHA7*	Aminoacid synthesis, neurite outgrowing, and neuronal migration	

miR-106b	Mouse neuroblastoma cell line and human hepatocyte	*ABCA1*	Cellular cholesterol efflux and A*β* production	[14]
